# Case Report: Gonio-endoscopy: a novel approach to minimally invasive glaucoma surgery in a glaucomatous eye

**DOI:** 10.3389/fopht.2023.1226316

**Published:** 2023-10-02

**Authors:** Kenji Matsushita, Rumi Kawashima, Noriaki Kanazawa, Shinichi Usui, Kohji Nishida

**Affiliations:** ^1^ Department of Ophthalmology, Osaka University Graduate School of Medicine, Suita, Japan; ^2^ Engineering Department, MACHIDA Endoscope Co., Ltd., Abiko, Japan; ^3^ Research & Development Center Department V, NIPRO CORPORATION, Kusatsu, Japan; ^4^ Integrated Frontier Research for Medical Science Division, Institute for Open and Transdisciplinary Research Initiatives, Osaka University, Suita, Japan

**Keywords:** gonio-endoscopy, minimally invasive glaucoma surgery (MIGS), microhook ab interno trabeculotomy, Glaucoma, cloudy cornea

## Abstract

**Background:**

The gonio-endoscope is a novel device for use during minimally invasive glaucoma surgery (MIGS) to treat glaucomatous eyes with cloudy cornea. The MIGS procedure requires a surgical gonioprism lens for direct visualization of the angle, intraoperative manipulation of the surgical microscope and the patient’s head position, and the patient’s eye without a cloudy cornea. In cases with cloudy corneas or limitation of neck movement, MIGS cannot be safely performed. Gonio-endoscopy facilitates clear visualization of the trabecular meshwork (TM) to perform MIGS safely and easily even in a patient with corneal opacities or limitation of neck movement with no additional MIGS procedures. We report the first case in which we performed the newly developed a 10,000-pixel high-resolution 23-gauge gonio-endoscopic operation.

**Case presentation:**

The patient was a 58-year-old man with Down syndrome who had secondary glaucoma bilaterally after cataract surgery and long-time use of a steroid for atopic dermatitis and allergic conjunctivitis. His left eye had a cloudy cornea after penetrating keratoplasty for keratoconus with severe corneal residual scarring after prior resolved corneal hydrops. When the intraocular pressure (IOP) in his left eye increased to 41 mmHg despite the maximum use of anti-glaucoma eyedrops, he was referred to our hospital. Anterior-segment optical coherence tomography showed an open angle. We developed a new gonio-endoscope (MACHIDA Endoscope Co., Ltd., Chiba, Japan and NIPRO CORPORATION, Osaka, Japan), the probe of which is bent appropriately to aid visualization of and access to the TM. After obtaining clinical approval from the government and our institution, we could safely perform a high-resolution 23-gauge gonio-endoscopy-assisted microhook ab interno trabeculotomy (μLOT). The IOP decreased to 10 mmHg and the visual acuity has been preserved with no major complications for 1 year postoperatively.

**Conclusion:**

This new technique of clear gonio-visualization using a gonio-endoscope might be helpful for a safe and easy μLOT in patients with cloudy corneas. This device can apply to other types of MIGS procedures and cases with pathological diagnoses of glaucoma that are difficult to treat.

## Background

Glaucoma is a leading cause of irreversible blindness. Because of the rapid increase in aging populations worldwide, the number of patients with glaucoma will increase dramatically (1). Although filtering surgery is the gold standard among glaucoma surgeries, it is an invasive procedure and associated with the risk of severe postoperative complications. On the other hand, minimally invasive glaucoma surgery (MIGS) has become increasingly available around the world. This is because MIGS has the following preferable qualities: ab interno microincision, minimal trauma, efficacy, high safety profile, and rapid recovery (2). This procedure requires intraoperative gonioscopy for direct visualization of the angle through a clear cornea and intraoperative manipulation of the microscope and the patient’s head. Therefore, it is difficult to perform in cases with corneal opacity or limitation of neck movement. An ocular endoscope facilitates the performance of gonio-surgery without the usual surgical techniques for MIGS, as mentioned (3–5).

We developed a new device, Gonio-endoscopy. The instrument is 23 gauge and bent to improve the safety and ease of use of angle surgery, unlike usual ocular endoscopes. This device can maintain clear visualization of the anterior chamber angle with a 10,000-pixel high-resolution image and create a 3-dimensional (3D) vision by using polarized glasses. Tanito developed a microhook and underwent ab interno trabeculotomy using the microhook, which was named microhook ab interno trabeculotomy (μLOT) and is now well known (6–8). In this report, we describe the gonio-endoscopy-assisted μLOT for a glaucoma patient with corneal opacity.

## Case presentation

**Figure 1:**
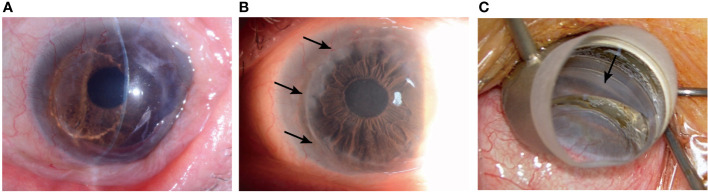
The left eye is treated with penetrating keratoplasty for keratoconus with severe corneal residual scarring after prior resolved corneal hydrops **(A)** and has a peripheral cornea opacification (arrows) **(B)**. The angle (arrow) cannot be detected through a surgical gonioprism lens **(C)**.

This study was performed in accordance with the Declaration of Helsinki and approved by the Ethics Committee of Osaka University Graduate School of Medicine (Institutional review board (IRB) Approval No. 17330-2). We obtained an informed consent from the patient. A 58-year-old man with bilateral secondary glaucoma after cataract surgery was referred to our hospital. He had been treated for an extended period with a steroid ointment and eyedrops for atopic dermatitis and allergic conjunctivitis. We could not precisely determine his iridocorneal angle or visual perimetry because of Down syndrome, but he had an open angle visualized by anterior-segment optical coherence tomography (CASIA2, Tomey, Nagoya, Japan) and glaucoma disc cupping bilaterally. His right eye had reached an intraocular pressure (IOP) of 46 mmHg using 3 kinds of anti-glaucoma eyedrops and had severe glaucoma disc cupping, although the visual acuity was unmeasurable. We performed Baerveldt glaucoma implant surgery on the right eye. Two years after that, the intraocular pressure (IOP) in his left eye increased to 41 mmHg despite the use of anti-glaucoma eyedrops: latanoprost, carteolol hydrochloride, brinzolamide, brimonidine tartrate. The visual acuity was 1.0 uncorrected log MAR. We diagnosed steroid-induced glaucoma and performed gonio-endoscopy-assisted μLOT under general anesthesia, because it was determined that we could not safely perform the surgery under local anesthesia. His left eye was treated with penetrating keratoplasty for keratoconus with severe corneal residual scarring after prior resolved corneal hydrops 21 years ago and had a graft rejection 6 years ago. The steroid therapy has made corneal opacity improve, but the peripheral corneal opacity and neovascularization have been worsening. (**Figures 1A, B**). Therefore, we could not detect the angle through a surgical gonioprism lens (**Figure 1C**). The conventional trabeculotomy is appropriate for the eye with cornea opacity or corneal endothelial damage, or after corneal transplantation. However, in this case, he was still in middle age with Down syndrome and the left eye was his dominant eye. So, we selected μLOT with soft shell technique, which preserves the conjunctiva and sclera intact for the future filtering surgery and has fewer sight-threatening complications, the short operation time, and the minimal invasiveness.

**Figure 2:**
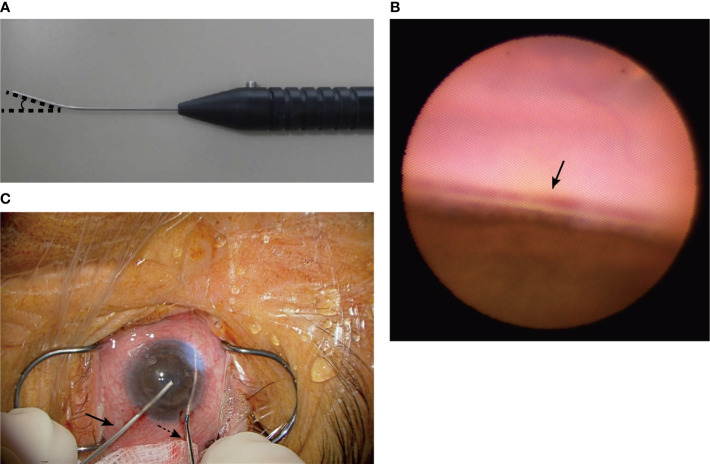
We developed the 23-gauge gonio-endoscope, which is bent 25 degrees and has a focus distance of 5 mm **(A)**. The gonio-endoscope facilitates clear visualization of the trabecular meshwork (arrow) **(B)**. We performed μLOT using the Matsushita ed. TMH (black dotted arrow) and gonio-endoscope (black arrow) and incised the trabecular meshwork nasally by 180 degrees **(C)**.

We developed a new 23-gauge gonio-endoscope (MACHIDA Endoscope Co., Ltd., Chiba, Japan and NIPRO CORPORATION, Osaka, Japan) with a light-emitting diode (LED) light source, the probe of which is bent 25 degrees and those focal length is 5 mm to help clear visualization of and access to the trabecular meshwork (**Figures 2A, B**), although the focal distance of currently used endoscope is 2 or 5 mm. We made two corneal side ports, injected an ophthalmic viscoelastic device into the anterior chamber, and inserted the gonio-endoscope and the Matsushita edition of the Tanito Micro-Hook trabeculotomy devices, i.e., the Matsushita ed. TMH (Eye Technology, Rayleigh, UK, and ME Technica, Tokyo, Japan) into each side port (**Figure 2C**). The intra-anterior chamber maneuvers were performed through a surgical microscope. The anterior chamber angle was visualized through the gonio-endoscope and the trabecular meshwork was incised nasally at 180 degrees. We successfully incised the trabecular meshwork by maintaining clear visualization (**Supplementary Video**). We did not need to change the position of the microscope or the patient’s face. During the visualization with the gonio-endoscope, the light from the endoscope and the surgical microscope were used. We can control the light intensity of the endoscope. The endoscope was pulled out and the viscoelastic device was removed through irrigation with an intraocular irrigating solution. Although he had temporal cystoid macular edema postoperatively, the outcome was good without severe hyphema, IOP spikes, or other complications. The IOP remained approximately 10 mmHg with glaucoma eyedrop: latanoprost, carteolol hydrochloride, pilocarpine hydrochloride, and the corneal transparency was maintained and the visual acuity was maintained at 1.0 uncorrected log MAR for one and a half years postoperatively.

## Discussion and conclusions

MIGS recently has been developed and requires a shorter surgical time, preserves the conjunctiva and sclera, and is associated with fewer sight-threatening complications, unlike filtering surgery, which traditionally has been the most performed. Various variations of the MIGS procedures have been developed: some are performed with an implantable device, some with special surgical equipment, and others without special equipment or implantation of a device (9). These procedures have lower intraocular pressure, less medication use, and fewer complications, but since these procedures are relatively new, the long-time safety and efficacy of MIGS are still being determined (10). However, in the worldwide, the interest and frequency of less invasive glaucoma surgeries have significantly increased (2, 11, 12).

A part of MIGS procedures (trabecular based procedures, ab interno trabeculotomy, Trabecutome, iStent, and Xen), require a gonioprism lens for direct visualization of the angle and intraoperative manipulation of the microscope and the patient’s head. Unfortunately, the cloudy cornea associated with secondary glaucoma or primary childhood glaucoma or limitation of neck movement according to the systemic disease or under general anesthesia may obscure visualization of the anterior chamber angle using an operating microscope and a surgical gonioscopy lens. An ocular endoscope facilitates the performance of any MIGS using a surgical gonioprism lens despite a cloudy cornea or limitation of neck movement. However, we must have a careful use of endoscope and perform MIGS procedures to avoid corneal endothelial damage.

In 1997, Medow was the first to report endoscopic goniotomy in infant with cloudy cornea and primary congenital glaucoma using a 20-gauge ocular endoscope (3). Afterward, in 2001, Joos reported a coaxial endoscopic goniotomy with a thin blood lancet wrapped tightly around a 20-gauge ocular endoscope (4). Recently, Nakao reported a surgical technique of transluminal trabeculotomy using a 23-gauge ocular endoscope for patients with limited conversion of his head (5). Previous ocular endoscopes have a straight probe. We developed a gonio-endoscope to be fit for the manipulation of the anterior chamber. Our gonio-endoscopy has a curved probe to get a clear visualization of the angle safely and easily and its focus distance is the best to maintain clear visualization of the anterior chamber angle. The depth-enhanced and parallax images acquired from the gonio-endoscope create a 3D image by using polarized glasses.

An endoscope coaxially coupled to a surgical instrument requires only 1 corneal incision, permits the continuous surgical view during the procedure, and is better to avoid damage to surrounding structures, such as endoscopic cyclophotocoagulation (ECP) probe (13, 14). Our gonio-endoscope is not a coaxial endoscope, so requires two corneal incisions. It may be necessary to get used to the procedure to get a stable endoscopic image or to avoid accidental damage to the surrounding structures during MIGS. In addition, the excessive bleeding during trabecular meshwork incision can make visibility through an endoscopy worse and corneal incision for a 23-gauge endoscope is required to make a suture. This is only a single case, so further we will need cases to evaluate the efficacy of the gonio-endoscope. However, the direct visualization of the iridocorneal angle is essential for diagnosing and managing glaucoma and for glaucoma surgeries, including childhood glaucoma. So, the gonio-endoscope could be extremely useful. Soon, the further minimally invasive surgery might be possible by using a 25-gauge gonio-endoscope. We believe that this new technique might assist in the development of innovations for MIGS procedures or glaucoma diagnosis in the future.

## Data availability statement

The original contributions presented in the study are included in the article/**Supplementary Material.** Further inquiries can be directed to the corresponding author.

## Ethics statement

The studies involving humans were approved by the Ethics Committee of Osaka University Graduate School of Medicine. The studies were conducted in accordance with the local legislation and institutional requirements. The participants provided their written informed consent to participate in this study. Written informed consent was obtained from the individual(s) for the publication of any potentially identifiable images or data included in this article. Written informed consent was obtained from the participant/patient(s) for the publication of this case report.

## Author contributions

KM had the substantial contributions to the conception and designed of the work. RK collected the data. RK and KM interpreted of data. RK and KM drafted the work and all authors substantively revised it. All authors contributed to the article and approved the submitted version.

## Supplementary material

Supplementary Video Gonio-endoscopy-assisted *ab interno* trabeculotomy using a microhook in the left eye.
